# How to Deal With an Unreconstructable Distal Radius Fracture

**DOI:** 10.7759/cureus.52487

**Published:** 2024-01-18

**Authors:** Angelos Assiotis, Adam Rumian, Harpal S Uppal, Clarence Yeoh

**Affiliations:** 1 Trauma and Orthopaedics, Lister Hospital, Stevenage, GBR

**Keywords:** intra-articular fracture distal radius, distal radio-ulnar joints, external ﬁxator as definitive fixation, unstable distal radius fractures, distal radius fracture management

## Abstract

The most prevalent long bone fracture is that of the distal radius, and it affects all age groups. These fractures can present after low-energy or high-energy trauma, and their configuration often varies depending on the mechanism of injury. Their management can be operative or non-operative, and the scientific literature is abundant in studies comparing these two treatment modalities. There is also a healthy scientific debate as to the indications that should guide surgery for these injuries. A male patient sustained a high-energy fracture to his distal radius and presented to our unit soon after the injury. His fracture presented significant surgical challenges due to its complexity. It was stabilised surgically, and the patient recovered good function after rehabilitation. This case aims to demonstrate a surgical treatment protocol and the relevant surgical considerations when dealing with significant injuries, such as the one presented in this paper, where traditional fixation techniques may not yield a satisfactory outcome.

## Introduction

Fractures of the distal radius are common injuries [1] and can be treated non-surgically or surgically. The functional outcomes following such treatment modalities have been reported and compared in the literature in adult patients [2-3] and although there is no universal consensus on surgical indications for these injuries, the current practice is that when a fracture is highly displaced and involves the intra-articular components of the distal radius, in an otherwise healthy and active patient, it should be treated with surgery.

Surgical options for managing distal radius fractures include manipulation under anaesthesia and casting, use of Kirschner wires and casting, non-locking plates, volar and dorsal fixed-angle locking plates, dorsal spanning plates, external fixators [4], and distal radius hemiarthroplasty [5]. These treatment options may also be used in combination. The aim is to restore the anatomical parameters of the distal radius, the radiocarpal, and the distal radio-ulnar joints (DRUJ) and, in doing so, allow the patient to rehabilitate as soon as feasible [4].

The case that we present in this paper is a very educational example of a very complex, high-energy injury that poses significant challenges in reconstructing the relevant anatomy. We used a number of techniques in the operative fixation of this fracture, and after appropriate rehabilitation, the patient has reached a satisfactory functional outcome. We present our surgical management and our decision-making process that guided our approach to this patient.

## Case presentation

**Figure 1:**
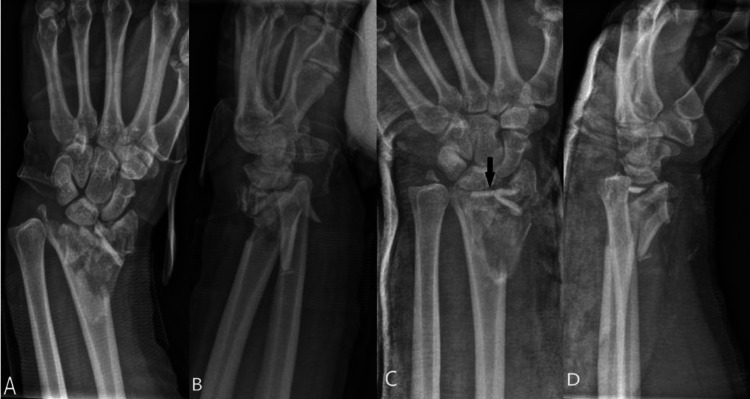
Antero-posterior and lateral radiographs on presentation A: Note the significant comminution and disruption of the radiocarpal joint surface. B: Note the dorsal subluxation of the radiocarpal joint and the significant comminution of the dorsal radial metaphysics. C: Anteroposterior radiograph after reduction. Note the significant comminution of the radial column and the large fragments of radial metaphysis present in the radiocarpal joint, as indicated by a black arrow. D: Lateral radiograph after reduction. The radiocarpal joint is still subluxed due to a lack of subchondral support, both palmarly and dorsally.

A 51-year-old male ambidextrous patient presented to our ED after a fall from his mountain bike, going around 30 miles an hour downhill. After he fell off his bicycle, he landed heavily on a tree stump with his wrist. He suffers from mild asthma and is a non-smoker. He runs his own business as a locksmith, and the use of both hands for fine tasks is crucial in his line of work. He presented with a closed, highly comminuted fracture of his distal radius and malalignment of the DRUJ. On presentation, he had hand numbness in the distribution of the median nerve. His distal radius fracture was manipulated in the ED, and the position improved as much as feasible before going into a plaster of Paris backslab (Figure 1A-1D).

**Figure 2:**
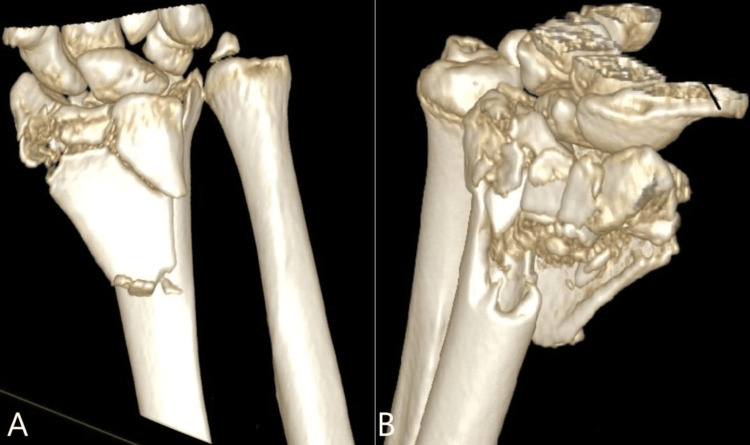
Three-dimensional reconstruction of the fracture A: Note the significant comminution of the volar metaphysis and articular surface of the distal radius. B: Note the comminution of the dorso-radial metaphysis of the distal radius. The fragments that originated from the fracture bed were incarcerated in the radiocarpal joint.

**Figure 3:**
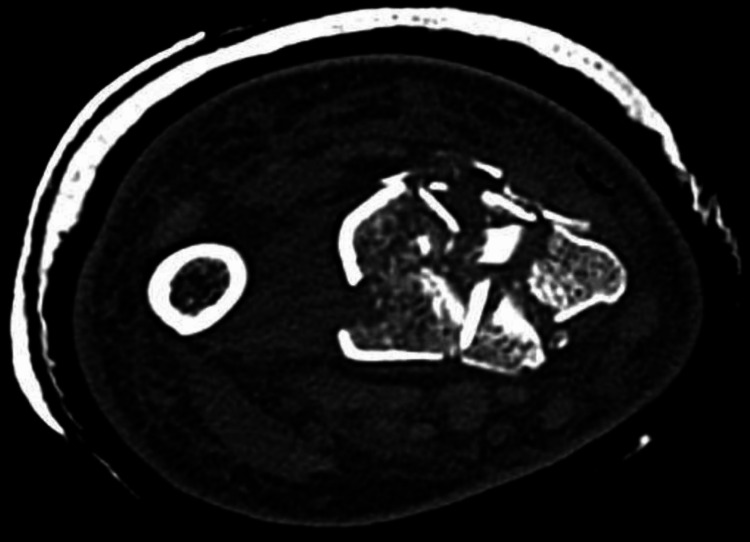
Axial computed tomography (CT) scan image This demonstrates the significant comminution of the distal radius and displacement of the lunate and scaphoid fossae.

He was admitted to the ward for elevation, as there was a concern regarding the potential development of compartment syndrome. In the following morning, his pain had much improved, and he still had mild carpal tunnel symptoms without neuropathic pain. The decision was made at that point to defer his surgical fixation to an appropriate specialist trauma list. A CT scan was arranged, which demonstrated the complexity of this injury in greater detail (Figure 2A-2B, Figure 3).

**Figure 4:**
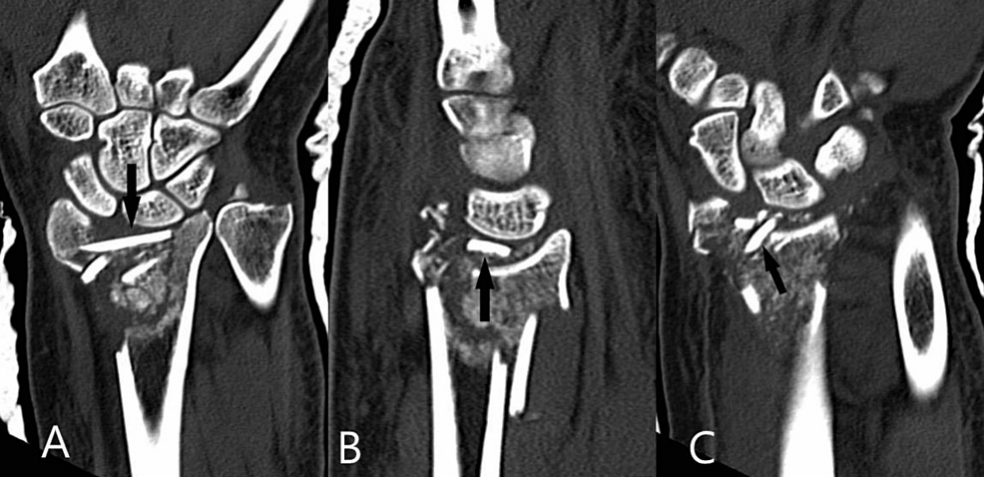
Computed tomography (CT) coronal and sagittal reconstructions A: This image demonstrates the displacement of cortical fragments within the radiocarpal joint, as indicated by the black arrow. These fragments were devoid of any soft tissue attachments. They were retrieved and reduced to the remaining dorsal distal radius. They were then stabilised with several suture knots. B: The black arrow demonstrates the displacement of the cortical fragments within the radiocarpal joint. The black arrow demonstrates the intra-articular position of the metaphyseal fragments.

Significant findings from this scan included disruption of the DRUJ, complete loss of contact between the scaphoid and lunate bones and the corresponding scaphoid and lunate fossae of the distal radius, and the displacement of cortical metaphyseal fragments in the radiocarpal joint through the primary fracture lines (Figure 4A-4C).

Prior to surgery, we arranged for an external fixator device to be available, volar and dorsal locking plates, and a dorsal wrist spanning plate system as well. The patient consented to all of the above and also to a decompression of his carpal tunnel. He was taken to the operating theatre four days after his presentation.

**Figure 5:**
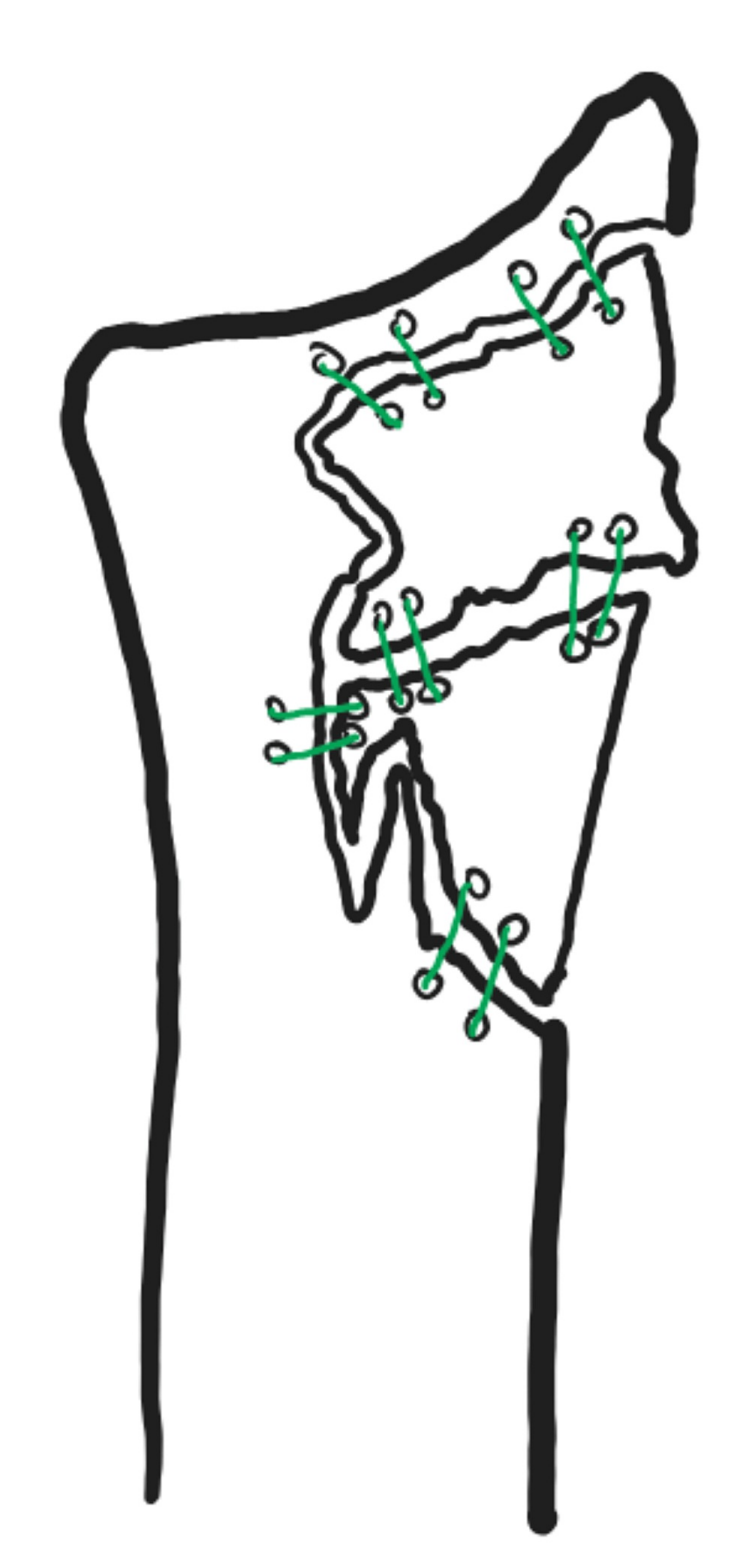
Schematic demonstrating the dorsal aspect of the distal radius The two main dorsal cortical fragments were retrieved from the radiocarpal joint, and multiple small perforations were made with a wire. These were placed in appropriate positions so that the fragments would then be approximated and held in place with multiple suture knots. Prior to this step, the metaphyseal defect in the distal radius was grafted with cancellous bone chips, creating a bed for the suture fixation construct.

The procedure was performed under a tourniquet, and we started with a decompression of the median nerve in the carpal tunnel, where the nerve was found to be intact but contused. We then carried on an extended flexor carpi radialis approach, as described by Orbay et al. [6]. Through this approach, we first reduced and buttressed the significant volar metaphyseal comminution with a volar locking plate. Distal screws were not inserted, as there was significant articular comminution, and they would not add to the stability of the construct because they would not purchase in bone and would not be able to offer significant subchondral support. Following this, a dorsal approach between the third and fourth extensor compartments was made. A posterior interosseous nerve neurectomy was carried out as the nerve was contused and incarcerated in comminuted fragments. Following this, several comminuted fragments of cortical bone were retrieved from the radiocarpal articulation. These fragments were placed in the appropriate position in the dorsal radial metaphysis, and from the proximal-to-distal direction, the dorsal radial metaphysis was reconstructed using size 2 FiberWire (Arthrex, Inc., FL, USA) sutures through drill holes as the method of fixation of these cortical fragments between them and onto the remaining radial shaft. In order to reconstruct a structurally sound metaphyseal area, onto which these cortical fragments would then rest, the radial metaphyseal defect was filled with freeze-dried cancellous allograft bone chips. The suture technique was not used to gain absolute stability but rather to reduce and stabilise loose cortical fragments in the correct orientation so that they would consolidate there (Figure 5).

**Figure 6:**
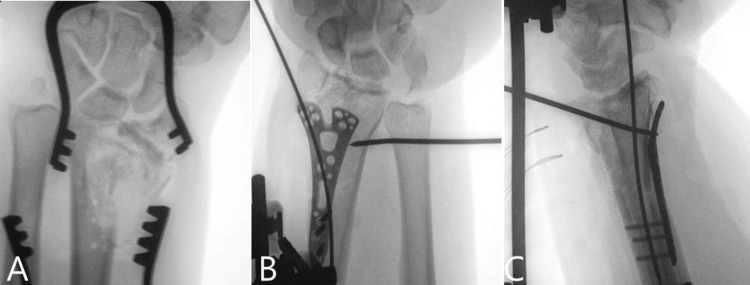
Intra-operative images of distal radius A: Demonstrating the placement of drill holes, which were used to reconstruct the radial metaphysis with sutures. B: This anteroposterior view shows the final outcome of stabilisation in theatre, with the external fixator attached. The transverse 2 mm wire was inserted to stabilise the distal radio-ulnar joint with the forearm in supination. C: Note the restoration of the joint surface and the correction of the dorsal tilt deformity.

At this point, the plan was to insert a dorsal spanning plate; however, this was not available at that time due to a logistical issue with our sterile services department. We, therefore, proceeded with the application of an external fixator device with 2.7 mm pins in the index finger metacarpal and 3.5 mm pins in the radial shaft, away from the zone of injury. Once constructed, this unilateral frame was used to improve reduction through ligamentotaxis. A 1.6 mm wire was introduced in the radial styloid fracture line, and the Kapandji technique [7] was used to improve the position of the radial styloid. At this point, the reduction was deemed satisfactory, and the external fixator construct was fully tightened. The DRUJ was found to be unstable, and a 2 mm transverse wire was introduced from the ulna to the radius, with the DRUJ reduced and the forearm in full supination. A decision was made at that point not to pursue an ulnar styloid fixation or additional stabilisation of the triangular fibrocartilage complex, as the forearm was very swollen and an additional incision was not in the patient’s best interests. The tourniquet time was 63 minutes, and the entire procedure lasted for 106 minutes. The tourniquet was released after the volar plate was inserted and before the external fixator was introduced. The patient was placed in an above-elbow cast in a position of forearm supination (Figure 6A-6C).

**Figure 7:**
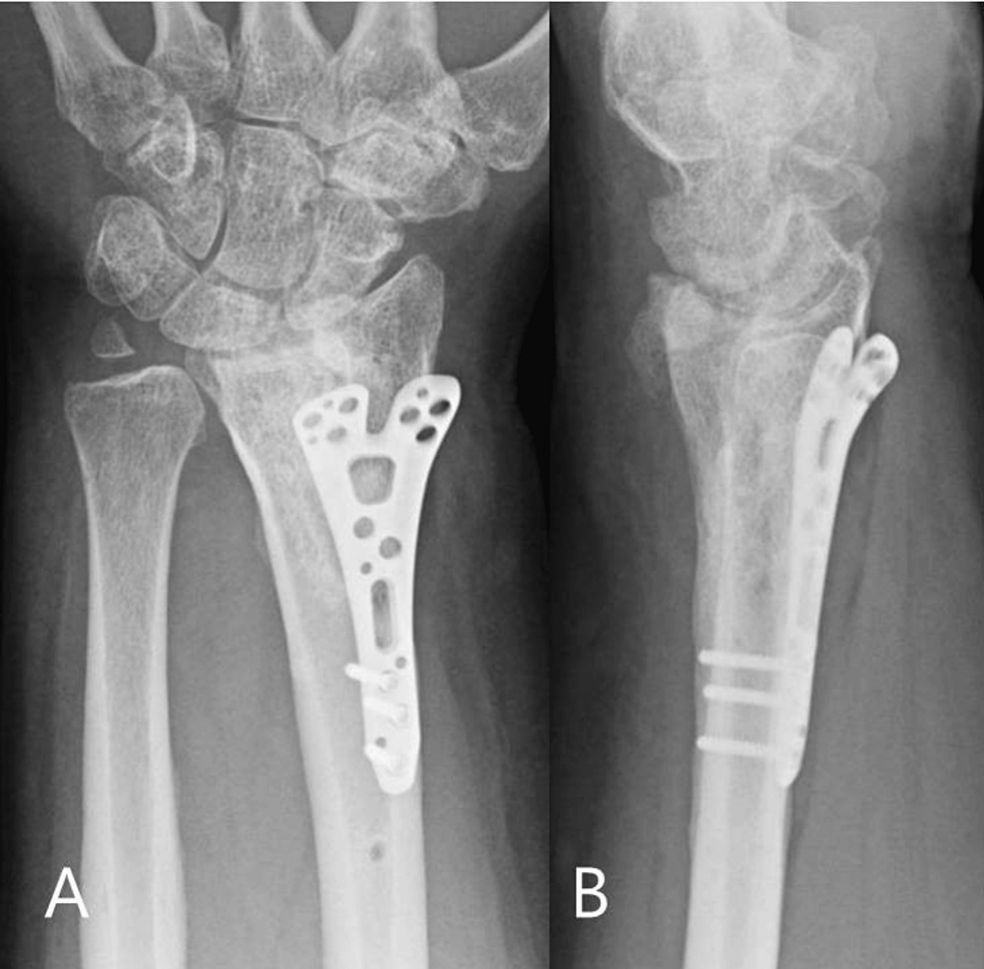
Radiographs at final follow-up A: Showing the restoration of the radial inclination and radial height and the consolidation of the fracture. B: Showing consolidation of the fracture and a small degree of residual dorsal tilt of the distal radius. This was due to the need for early removal of the external fixator as the pin sites became contaminated.

The patient was reviewed in our fracture clinic on a weekly basis. His median nerve symptoms were fully resolved by the first week after surgery. The external fixator construct was re-tightened every week, and the pins were removed four weeks after surgery, as the pin sites had become infected. The pin site infection was superficial and was identified three weeks after surgery. He was prescribed oral antibiotics for one week, and then the external fixator construct was removed. The DRUJ pin was also removed at that stage, and the patient spent another two weeks in a cast (total cast immobilisation was six weeks). He underwent prolonged and intensive physiotherapy and has regained a significant proportion of his pre-injury function. He has returned to work as a locksmith. Two years after the surgery, his patient-rated wrist evaluation score was 23, and his QuickDASH score was 10. Both of these outcome measures have been validated for use in distal radius fractures [8]. Radiographs at two years demonstrated complete consolidation of the fracture, with a reduced DRUJ and restored radial height and radial inclination. There was a degree of dorsal tilt of the articular fragment, most likely due to the early removal of the external fixator, which became necessary after the pin sites became contaminated (Figure 7A-7B).

**Figure 8:**
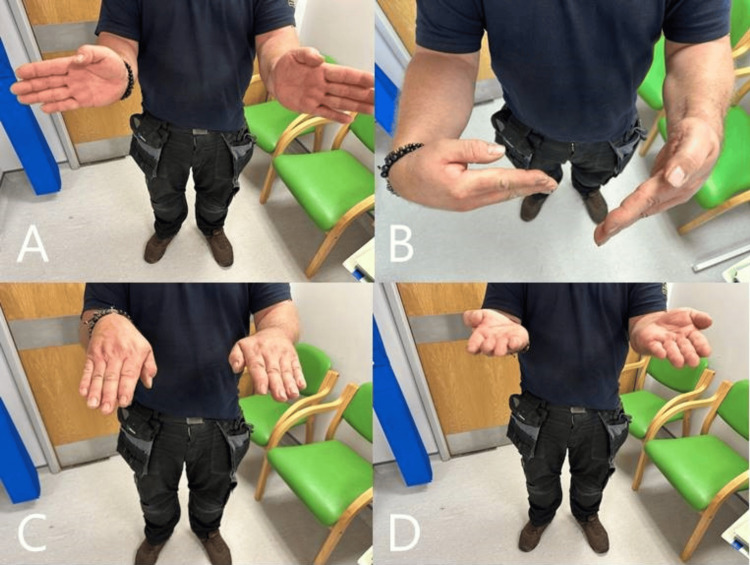
Clinical images at final follow-up A: Extension of the left (injured) wrist, compared to the right (uninjured) side. B: Flexion of the left wrist, compared to the right side. C: Pronation of the left wrist, compared to the right side. D: Supination of the left wrist, compared to the right side.

A goniometer was used in the clinic to assess his range of movement. His supination was 80 degrees from neutral, his pronation was 70 degrees, his extension was 50 degrees, and his flexion was 25 degrees. He was able to form a full fist and remains satisfied with his functional outcome (Figure 8A-8D).

## Discussion

Distal radius fractures are prevalent, and their surgical management is often technically straightforward, either through the use of wires or plates. When considering volar locking plates for these injuries, our team has already published our technique of gaining excellent temporary reduction with wires before applying the plate [7]. In the case of intra-articular fractures of the distal radius, the technical objective is usually anatomical reduction of the main fragments and absolute stability of the fixation construct. In the case of our patient, the goal of absolute stability could not be achieved, and as such, this was not part of our surgical plan.

Some distal radial fractures, however, can be very complex and may pose significant technical challenges, where the frequently used reduction and stabilisation techniques may not work and the aim of absolute stability cannot be achieved. Our case is one such example, where alternative techniques need to be considered and complete anatomical reduction of the several fragments is not possible.

In terms of our surgical plan, the goal was to recreate the best possible articular block and then to achieve a union between the articular block and the metaphysis of the radius, as this outcome would allow us to consider further reconstructive options later down the line, such as partial or total radiocarpal arthrodesis. Our initial plan was to use a dorsal spanning plate, but we were not able to do that as the implant was not available at the time of surgery. Instead of a plate, we used an external fixator construct in order to provide adequate ligamentotaxis. The rationale behind our decision to do that was the severity of the injury and the timing of surgery. The patient was taken to the theatre four days following the presentation because this was the first available upper limb trauma list that the unit had available. The British Orthopaedic Association has published its standards for trauma for distal radius fractures and intra-articular fractures, which should be operated on within the first three days after presentation. The authors decided to proceed with the operation instead of rescheduling because we also agree that such significant injuries should be dealt with as soon as possible. We believe that the outcome justifies our decision. Following rehabilitation, he will not require further procedures, as he is satisfied with his functional outcome.

Alternative surgical options, such as cast immobilisation and wire fixation, were not appropriate here, as they would result in a very poor functional outcome. In this case, we could not use our published technique to reduce fractures of the distal radius [7] prior to plate application, as he did not have an intact metaphysis for the wires to push against. Plating for the dorsal distal radius was not an option either, as the significant comminution he had meant that there could be no buttress function to the proposed dorsal fixation construct. Fragment-specific fixation with locking plates for the radial and intermediate columns was not appropriate here due to the comminution of the bone. A primary wrist hemiarthroplasty, which is a relatively new surgical option for severely comminuted distal radius fractures [9], could not be used as the patient had significant metaphyseal comminution that could not provide the appropriate support for a hemiarthroplasty implant. Furthermore, he is a young, high-functioning patient with a manual job, and wrist hemiarthroplasties have been mainly studied in elderly patients with lower functional demands. Ideally, we would have preferred to use a dorsal spanning plate as opposed to an external fixator, as this would allow us to retain the fixation for a longer period of time, but as this was not available on the day, we decided to proceed with the external fixator. Finally, an important point that should be made is that during the reconstruction of the distal radius, we ensured that fragments that had some soft tissue attachment to them were not further devascularised through rigorous mobilisation. We feel that this had a significant effect on the favourable outcome in this case, where the fracture united and consolidated successfully.

This case presents a common fracture in a very uncommon presentation, with significant surgical challenges. We aim to describe a surgical plan that can yield a satisfactory functional outcome for these seemingly ‘unreconstructable’ fractures. We would also like to raise awareness that such injuries are better managed in units that have experience with significant and complex upper limb trauma and the full provision of hand therapy services.

## Conclusions

Distal radius fractures that present with significant comminution may not be amenable to the standard treatment modalities. A specific key point in managing such complex injuries is careful pre-operative planning based on a CT scan in order to plan appropriate surgical approaches, the order of reconstruction, and the necessary surgical equipment. Another important point is that any trauma unit dealing with complex trauma should have an external fixation system available on the shelf in case this becomes necessary, as it did in our presented report.

The general principles in managing highly comminuted distal radial fractures include early surgery after presentation, the use of ligamentotaxis to recreate the gross anatomy of the region, and the consideration of options such as wires, sutures, and limited use of plates in order to reconstruct the articular component and allow that to unite on the metaphysis of the distal radius.
